# Plasma-treated polylactic acid films combined with ε-polylysine-chitosan coatings for fresh pork packaging

**DOI:** 10.1016/j.fochx.2025.103353

**Published:** 2025-11-30

**Authors:** Li Yana, Zhou Guorui

**Affiliations:** Wuhan Polytechnic University, Wuhan, China

**Keywords:** Antibacterial, Bilayer, Biodegradable, Food packaging

## Abstract

Due to threats of environmental pollution and food safety, the polylactic acid (PLA) was treated by plasma prior to coating chitosan (CS)/ε-polylysine (ε-PL) to expand the application in active packaging. Air plasma significantly enhanced the compatibility of PLA with coating to obtain a closely integrated bilayer ε-PL-CS/PLA. The coating endowed antibacterial activity of PLA from 0 % to 57.06 % for *E. coli* and 33.17 % for *S. aureus,* and decrease its oxygen barrier properties dramatically from 127.33 to 1.73 cm^3^/m^2^·24h·0.1 MPa. Increasing the ε-PL content enhanced the elongation at break, hydrophilicity and bioactivity of ε-PL-CS/PLA, while reduced the tensile strength and moisture barrier properties. In food applications, the ε-PL-CS/PLA films effectively delayed the change in redness, pH, TVC and TVB-N during storage, prolonging the shelf life of pork from 2 d (PLA) to 8 d (10 %-ε-PL-CS/PLA). This study confirms the potential of modified-PLA films with excellent antibacterial properties for active packaging.

## Highlights

• Enhanced compatibility between PLA and ε-PL/CS coating by plasma treatment.
• Improved oxygen barrier and antibacterial properties of PLA by functional coating.
• Antibacterial properties of bilayer films increasing with increase of ε-PL content.
• Extending shelf life of pork from 2 d (PLA) to 8 d (10 %-ε-PL-CS/PLA).

## 1 Introduction

Amid growing global emphasis on sustainable development, food safety has become an increasingly prominent concern among consumers (Li, Qi, & Ahmad, 2025). The development of biodegradable packaging materials with active preservation capabilities has emerged as a critical research direction in the food industry (Nampoothiri, Nair, & John, 2010; Siracusa, Rocculi, Romani, & Dalla Rosa, 2008; Bélard, Poncin-Epaillard, Dole, & Avérous, 2013； Zandén et al., 2012).

Among biodegradable material systems, natural polysaccharides have attracted considerable attention due to their excellent biocompatibility and degradability (Aziz et al., 2024). Chitosan (CS), a natural cationic polysaccharide, demonstrates outstanding film-forming capacity and inherent antibacterial characteristics. However, its inadequate mechanical strength and antibacterial properties limit its standalone application in food packaging.

The ε-polylysine (ε-PL) is a kind of natural antimicrobial peptide, which has the characteristics of wide antibacterial spectrum, good water solubility, high safety, good thermal stability, etc. Therefore, ε-PL has received a lot of attention as a food preservative and in food packaging as an antibacterial enhancer to chitosan. Liao et al. (Liao et al., 2023) demonstrated the effectiveness of an active packaging film based on CS/ε-PL-grafted bacterial cellulose in delaying the spoilage of tilapia and inhibiting bacterial growth. The composite film prepared by CS and ε-PL was used as the coating to obtain the food packaging film material with green environmental protection function, in which the addition of ε-PL as an antibacterial agent can effectively inhibit the growth and reproduction of microorganisms (Geornaras and Sofos, 2005; Geornaras, Yoon, Belk, Smith, & Sofos, 2007; Pandey and Kumar, 2014; Shih, Shen, & Van, 2006; Shima, Matsuoka, Iwamoto, & Sakai, 1984; Zhao, Huan, Yang, Chen, & Yao, 2025).

Polylactic acid (PLA), another prominent biodegradable polymer, offers favorable mechanical properties and water resistance. Nevertheless, that it has no antibacterial activity and poor gas barrier properties have constrained its advancement in food packaging applications (Liu, Cui, et al., 2021). Recent research has focused on integrating the advantages of PLA with CS through composite systems. However, Vasile et al. (Vasile et al., 2018) observed that CS/PLA films prepared via extrusion exhibited brittle fracture and reduced elongation at break. Mohamad et al. (Mohamad et al., 2021) found that cast-prepared CS/PLA films demonstrated diminished mechanical properties, poor hydrophilicity, and inadequate compatibility. Our team's preliminary research found that the low surface energy and weak polarity of PLA create significant thermodynamic incompatibility with hydrophilic chitosan, resulting in inadequate interfacial adhesion and hindering the formation of stable, uniform bilayer structures (Li et al., 2020). Thus the interface compatibility is one of the biggest challenges to develop composite materials that combine the mechanical advantages of PLA with chitosan based bioactive substances. A possible strategy is to selectively modify the surface of a material using different modification techniques.

In the past decade, the use of nonthermal plasmas for surface modification has been a rapidly growing research field. Plasma technology belongs to dry state processing, which is energy-saving, water-saving, efficient and clean, and has no environmental pollution (Desmet et al., 2009). Previous research has established plasma treatment as an effective method for enhancing surface polarity and wettability of polymeric materials by introducing a large number of polar functional groups (such as hydroxyl -OH, carboxyl -COOH, and carbonyl C=O) onto the film surface through bombardment by high-energy particles in the plasma and subsequent chemical reactions (Desmet et al., 2009). Leluk et al. (Leluk, 2023) demonstrated that surface modification of low-density polyethylene (LDPE) and polylactic acid (PLA) films using various plasma sources (argon, air, nitrogen, and oxygen plasma) converted LDPE surfaces from hydrophobic to hydrophilic characteristics. A substantial reduction in water contact angle was also observed in plasma-modified PLA specimens, with initial values of 60° decreasing to approximately 10° following argon, air, and nitrogen plasma treatments. Morent et al. (Morent et al., 2010) reported that air plasma treatment reduced the water contact angle of PLA films from 75° to about 58°. In research conducted by Vergne et al. (Vergne et al., 2011), atmospheric pressure nitrogen plasma treatment was utilized for PLA surface modification, resulting in significantly enhanced surface hydrophilicity with the water contact angle sharply declining from 78.8° to 42.7°. However, to date, no reports exist on plasma treatment for forming a dual-layer composite film by combining PLA with CS.

**Fig. 1:**
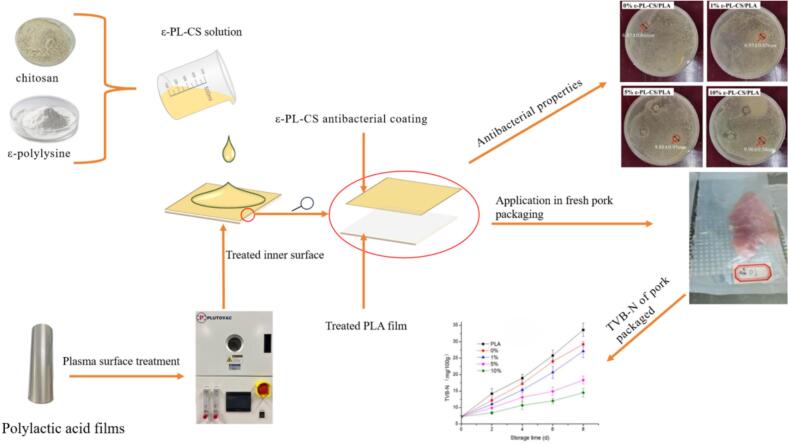
Plasma-treated PLA films combined with ε-polylysine-chitosan coatings for fresh pork packaging.

This study firstly applied air plasma treatment to PLA surfaces, followed by coating with CS and ε-PL blends, preparing ε-PL-CS/PLA bilayer films. The effects of plasma treatment on PLA surfaces were investigated, and the properties of the ε-PL-CS/PLA films, including mechanical properties, water vapor transmission rate, oxygen transmission rate, water contact angle, and antibacterial performance, were evaluated. Finally, the application effect of ε-PL-CS/PLA films in fresh pork packaging was examined. The graphical abstract is shown in Fig. 1.

## 2 Materials and methods

### 2.1 Materials

The PLA film was purchased from Zhuhai Hengqin Huizefeng Packaging Materials Co., LTD. The CS and ε-PL was purchased from Sinopharm Chemical Reagent Co., LTD. and Zhejiang Maya Reagent Co., LTD., respectively. The bacteria of *S. aureus* (ATCC29273) and *E. coli* (ATCC25922) were derived from the School of Food Engineering, Wuhan Polytechnic University. The pork was purchased from Zhongbai Supermarket in Wuhan, China. All chemical reagents are analytically pure and all formulation solvents are deionized water.

### 2.2 *Preparation of* ε*-PL-CS/PLA films*

#### 2.2.1 Plasma treatment of PLA

Adapted from the methods of reference, PLA film was cut into a 120 × 120 mm square sample, then pasted around it in a glass plate with transparent adhesi*v*e and put into a plasma cleaner for 1 min, 3 min, 5 min, 10 min, 15 min and 20 min respectively (Li, Li, et al., 2023). The power was 120 w and the plasma treated gas was air.

#### 2.2.2 *Preparation of* ε*-PL-CS antibacterial coating*

First, 2 g CS was dissolved in 100 mL 1 % (*v*/v) acetic acid solution and stirred at room temperature at 800r·min^−1^ for 4 h. Then, based on the previous study of our group, 0 %, 1 %, 5 %, and 10 % (according to the *w*/w of CS) ε-PL were added to their respective CS solutions, and stirred at 800 r·min^−1^ for 4 h to obtain 0 %, 1 %, 5 %, and 10 % ε-PL-CS solutions (Zhang, Han, & Zhou, 2024).

#### 2.2.3 *Preparation of* ε*-PL-CS/PLA composite films*

The PLA film treated by plasma for 5 min was chosen for composite film fabrication due to the best hydrophilicity and surface stability discussed in part 3.1. The ε-PL-CS solution (3 mL) was poured on PLA film, and applied evenly with a coating rod. The composite film was then dried in oven at 35 °C for 4 h. The finished ε-PL-CS/PLA composite films with different ε-PL concentrations of 0 %, 1 %, 5 %, 10 % were marked as 0 %-ε-PL-CS/PLA, 1 %-ε-PL-CS/PLA, 5 %-ε-PL-CS/PLA, and 10 %-ε-PL-CS/PLA, were prepared by the same method as above steps.

### 2.3 *Characterization of* ε*-PL-CS/PLA composite films*

#### 2.3.1 Thickness

Thickness measurement was carried out with a thickness gauge (GH-D, Guangzhou Biogi Packaging Equipment Co., LTD., Guangzhou, China). The final value of the measurement result is the average of 3 randomly selected points on the surface of the films.

#### 2.3.2 Mechanical property

The tensile strength and elongation at break of the sample were measured by an electronic universal testing machine (UTM4104, Shenzhen SyZH Co., LTD., Shenzhen, China). According to the method mentioned by Qi et al. (Qi and Li, 2024), the film was cut into a sample of 100 mm × 15 mm, the initial distance was set to 50 mm, and the measurement rate was 100 mm/min. Each group measured 3 samples and took their average value as the final data.

#### 2.3.3 Water contact angle

The water contact angle (WCA) of the film was determined by a contact Angle tester (JCY-3, Shanghai Fangrui Instrument Co., LTD., Shanghai, China). According to the method provided by Zhao et al. (Zhao et al., 2022), the test water drop was 2 μl, and the WCA was recorded 5 s later. The experiment was repeated 3 times and its average value was taken.

The test is used for three parts: 1) To determine the surface WCA of ε-PL-CS/PLA with different ε-PL content; 2) The influence of different bombardment time on the surface WCA of PLA film was investigated. 3) The PLA film was placed for 28 days, and the WCA was measured every day, so as to investigate the durability of the film surface polarity.

#### 2.3.4 Water vapor transmittance

The water vapor transmittance (WVT) of the sample was determined using the water vapor transmittance tester (W3/031×, Jinan Languang Electromechanical Technology Co., LTD., Jinan, China). According to the method mentioned by Qi et al. (Qi and Li, 2024). The film samples were cut into 74 mm diameter circles, placed in triplicate in a permeation cell containing deionized water (90 % relative humidity at 28 °C), weighed, placed in a desiccator, and averaged using a water vapor transmission rate tester. Three parallel samples were measured each time, and the final data were averaged.

#### 2.3.5 Oxygen transmittance

The oxygen transmittance (OT) of the sample was determined by the differential pressure gas transmittance tester (N530L, Guangzhou Biji Packaging Equipment Co., LTD., Guangzhou, China). According to the method mentioned by Li et al. (Li, Liu, et al., 2023), three parallel samples were measured each time, and the final data were averaged.

#### 2.3.6 Morphology

The scanning electron microscopy (SEM; JSM7200F, JEOL, Tokyo, Japan) was used to investigate (Zhang et al., 2024): 1) the cross-section of PLA film coated with chitosan (PLA/CS composite film) to study the compatibility between PLA-CS interface before and after plasma treatment for PLA substrate; 2) the surface morphology of ε-PL-CS/PLA with different ε-PL content.

#### 2.3.7 Antibacterial properties

The antibacterial activities of the films against *Staphylococcus aureus* (*S. aureus*) and *Escherichia coli* (*E. coli*) were tested according to the method described by Li (Li, Li, et al., 2023). The bacteria suspension (100 μL) was poured onto the agar medium (20 mL) and coated uniformly. The film samples of 5 mm diameter disks were placed on the inoculated plates with appropriate intervals, then were incubated at 37 °C for 24 h. The diameter of inhibition zones formed was measured by calipers, and which was used as one of indicators of the antimicrobial activity.

On the other hand, after incubation of the films with the bacterial suspension (10^6^–10^7^ CFU/mL) for 24 h, 1 mL of the bacterial culture solution was aseptically withdrawn and subjected to a series of tenfold serial dilutions using sterile normal saline as the diluent. Each dilution was thoroughly mixed and plated on nutrient agar using the spread plate method. The survival bacteria was pictured and counted to calculate the antibacterial activity of films using the formula (1).

(1) $$\text{Antibacterial activity}\left(\%\right)=\left(\frac{N_{1}-N_{0}}{N_{0}}\right)\times 100\%$$

where N_0_ and N_1_ are colony counts for the uncoated PLA and coated PLA samples, respectively. All assays were conducted in triplicate.

#### 2.3.8 Fourier transform infrared

Fourier transform infrared (FTIR) spectra of the films were measured using a Fourier transform infrared spectrometer (WQF-530 A, Beifen Ruili Analytical Instrument Co., Ltd., Beijing, China) in the range of 4400–400 cm^− 1^ with a resolution of 4 cm^− 1^ (Vergne et al., 2011).

### 2.4 Application in fresh pork packaging

The ε-PL-CS/PLA films were heat sealed into bags then to pack fresh pork samples (10 g) with ε-PL-CS as the inner layer to contact pork and the PLA was used as the control. All the pork samples were stored at 4 °C for 8 days and taken out every two days for quality test including pH, total viable count (TVC), redness (a*) and total volatile salt nitrogen (TVBN) according to the reference (Li et al., 2025). Total volatile basic nitrogen (TVB-N) values of samples were measured by a semi-micro Kjeldahl distillation method according to the Chinese standard GB5009.228–2016. Total viable counts (TVC) of samples were measured by plate count method. The pH value and redness (a*) of samples were measured using a digital pH meter (pH-100PRO, Shanghai Lichen Bangxi Instrument Technology Co., LTD., Shanghai, China) and a colorimeter (CR-10, Konica Minolta, Japan), respectively (Liu, Cui, et al., 2021).

### 2.5 Statistical analysis

Multiple samples were examined and data results reported as mean ± standard deviation (SD). SPSS22.0 was used for analysis of variance to evaluate the difference between single factor and level. Duncan multiple interval comparison test was used to determine which groups were significantly different from the other groups (*p* < 0.05) (Li, Wu, Li, Wang, & Chen, 2022).

## 3 Results and discussion

### 3.1 Effect of plasma treatment on PLA

#### 3.1.1 Water contact angle

The water contact angle (WCA) refers to the angle of the tangent line between the solid-liquid interface and the gas-liquid interface at the triple point, and reflects the hydrophobicity of the film surface. Generally, the WCA being greater than 65° indicates a hydrophobic surface, while being less than 65° is a hydrophilic surface (Valencia, Luciano, Lourenco, & do Amaral Sobral, P. J., 2018).

**Fig. 2:**
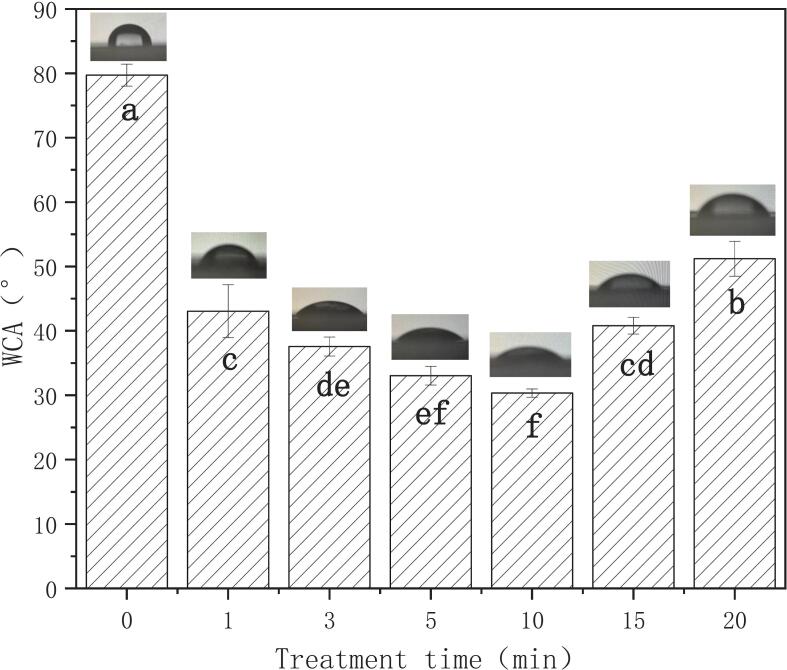
WCA of PLA films as a function of plasma treatment time, in which, different lowercase letters indicate significant differences (*p* < 0.05).

As can be seen from Fig. 2, the WCA of PLA surface was dramatically reduced after plasma treatment compared with original PLA. The WCA of PLA film decreased from 79.72° to 43.05° with treatment for 1 min, and the maximum reduction of the WCA of PLA film was 30.33° after plasma treatment for 10 min, which was no significant with 5-min treatment. After a longer treatment time for 15 min or 20 min, the WCA of PLA film decreased to 51.02°. Heidemann et al. (Heidemann, Dotto, Laurindo, Carciofi, & Costa, 2019) found that the WCA of PLA film decreased significantly after plasma treatment under atmospheric pressure and air conditions and the treatment time of 15 min or more caused no improvement on the surface WCA of PLA film, which was similar to the results observed in this study. The significant decrease of WCA after plasma treatment indicates that the wettability of the film surface increases compared with original PLA. The notable decrease in water contact angle (WCA) following plasma treatment indicates improved surface wettability of the film relative to untreated PLA. This enhancement can be attributed to both increased surface roughness induced by plasma treatment and the formation of radicals that interact with atmospheric oxygen species, leading to the incorporation of highly hydrophilic functional groups including hydroxyl (-OH), carboxyl (-COOH), and carbonyl (C=O) groups onto the polymer surface. From the Fig. 2, the treatment of 5 min or 10 min was the best.

#### 3.1.2 Surface stability

**Fig. 3:**
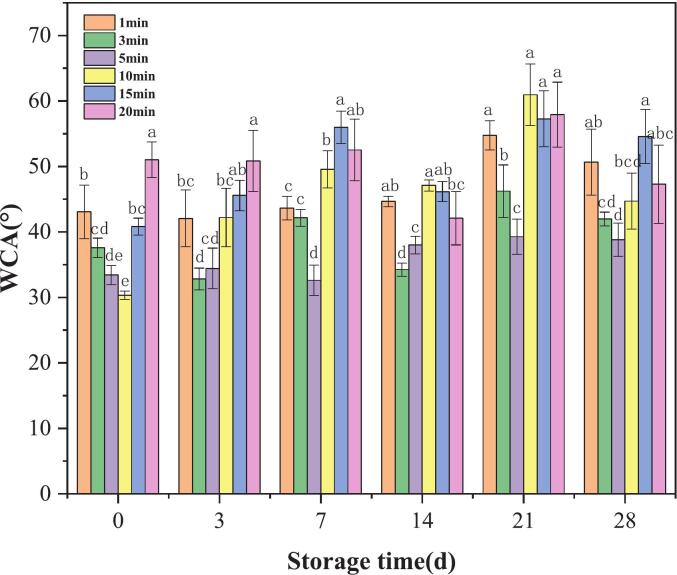
WCA of PLA films as a function of storage time, in which, different color of the columns means different treatment time as indicated in the figure, and different lowercase letters for the columns with same color indicate significant differences (*p* < 0.05).

Due to storage, plasma-activated PLA gradually loses its hydrophilicity, and its change process depends on the plasma treatment time shown in the Fig. 3, which is consistent with the phenomenon discovered by Izdebska-Podsiadły et al. (Izdebska-Podsiadły and Dörsam, 2021). This hydrophobic recovery is due to the removal of polar groups from the surface or their movement into the body of the polymer.

It was found that for the sample treated for 5 min, the WCA changed the least in the storage time of 28 d, while the sample treated for 10 min or more has a large degree of WCA change. Thus the the preparation of subsequent double-layer films was carried out using a 5-min treatment for PLA substrate due to the nice stability as well as surface hydrophobicity. However, regardless of how long the samples were stored, they retained some of their hydrophilic characteristics (Morent, De Geyter, Desmet, Dubruel, & Leys, 2011).

#### 3.1.3 Compatibilization of PLA surface to chitosan

**Fig. 4:**
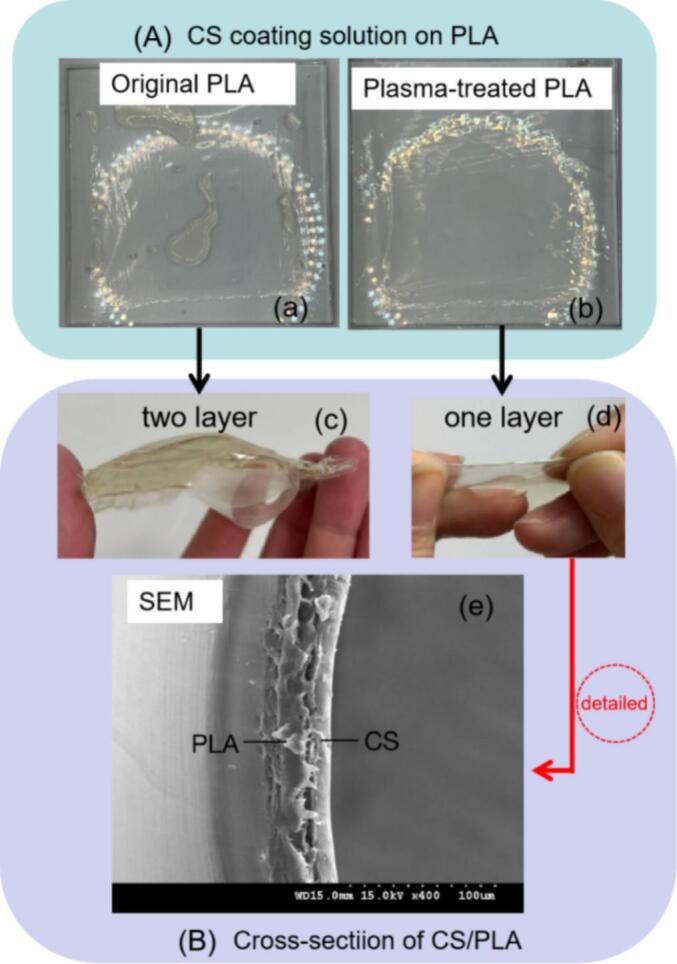
Compatibilization of PLA surface to chitosan.

According to the result of water contact angle and surface stability, the treatment time of 5 min was used to prepared the composite films. To verify the effectiveness of surface modification for PLA, the CS solution was coated on PLA before or after plasma treatment prior to observe the aggregation state of CS solution on PLA and the cross section of CS-PLA. The result was shown in Fig. 4.

It can be seen from Fig. 4a that when the chitosan solution was poured on the PLA surface, it gathered into large blocks on PLA without plasma treatment, and they survived in a two-layer film after drying shown in Fig. 4c, however, the solution blocks disappeared and the solution was uniformly coated on the PLA surface treated by plasma (Fig. 4b) to form CS/PLA composite film appearing to be a single-layer structure to the naked eye (Fig. 4d) with a tightly integrated interface between PLA and CS as shown in SEM picture in Fig. 4e. That indicates the compatibilization of PLA surface to chitosan was effectively improved by plasma treatment, which is the key to prepare the ε-PL-CS/PLA films in the next step (Jordá-Vilaplana, Fombuena, García-García, Samper, & Sánchez-Nácher, 2014). Karakurt et al. (Karakurt et al., 2022) also confirmed that plasma treatment improves the adhesion between CS and PLA films.

### 3.2 *Characterization of* ε*-PL-CS/PLA films*

#### 3.2.1 SEM

**Fig. 5:**
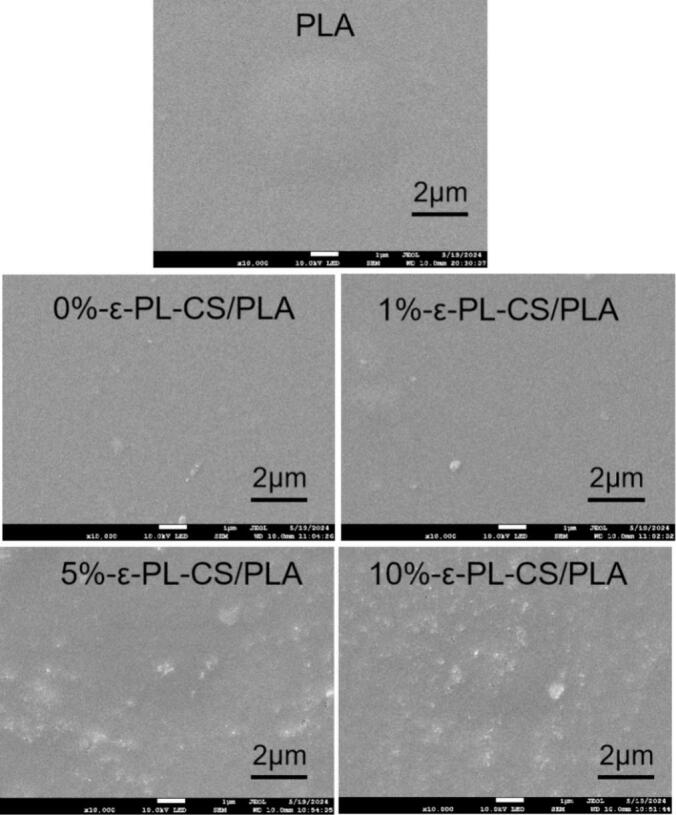
Surface SEM of composite films.

As shown in Fig. 5, the surface of the PLA film initially exhibits a relatively smooth morphology. After coating with 0 % and 1 % ε-PL–CS, the surface remains largely smooth, with only minor protrusions that are likely attributable to ε-PL particles. When coated with ε-PL–CS at concentrations above 5 %, a significant increase in surface roughness is observed, presenting a distinctly undulating morphology. This morphological change arises primarily from strong electrostatic interactions and hydrogen bonding between the abundant primary amino groups in the ε-PL chains and the hydroxyl/amino groups on the chitosan chains, leading to localized cross-linking and aggregation. Concurrently, the increased ε-PL content enhances its tendency to self-aggregate, forming microscopically phase-separated structures during the coating and drying process, which results in visible aggregates on the surface. Notably, even at high ε-PL concentrations, no significant pores, cracks, or interfacial delamination are observed on any of the ε-PL–CS/PLA composite film surfaces. This indicates excellent interfacial compatibility between the ε-PL–CS coating and the plasma-treated PLA substrate (Liu et al., 2025; Qi et al., 2025).

#### 3.2.2 Thickness

Thickness is an important index that affects the light transmittance, mechanical strength and water vapor transmittance of food packaging film. The molecular composition and intermolecular force are one of the factors affecting the thickness of the composite film. As shown in Table 1, the thickness of original PLA was 61.05 μm. With the increase of ε-PL content, the sample thickness of composite films increased significantly (*P* < 0.05), from 61.67 μm to 67.43 μm, and the thickness of composite films with different content of ε-PL was significantly different. This result may be influenced by the interaction of the amino group in CS with the carboxyl group in ε-PL, which increases the molecular spacing in the polymerized chain (Liu et al., 2020). Yu et al. (Yu, Liu, and Huo, 2019) also found that CS combined with ε-PL improved its thickness.

**Table 1:** Thickness, mechanical properties, WCA, WVP, OP and antibacterial activity of ε-PL-CS/PLA composite films.

Film	Thickness (μm)	TS(MPa)	EB(%)	WCA(°)	WVT(×10^−12^ g·m^−1^·s^−1^·Pa^−1^)	OT(×cm^3^/m^2^·24h·0.1 MPa)	Antibacterial activity (%)	
							*E. coli*	*S. aureus*
PLA	61.05 ± 1.47^cd^	21.56 ± 1.50^d^	82.13 ± 4.62^b^	79.72 ± 1.70^d^	1.52 ± 0.01^b^	127.33 ± 5.46^c^	0	0
0%-*ε*-PL-CS/PLA	61.67 ± 0.82^c^	18.24 ± 0.17^a^	80.32 ± 4.93^b^	55.05 ± 2.18^a^	1.49 ± 0.07^b^	2.03 ± 0.05^ab^	26.96 ± 0.11^bc^	3.59 ± 0.03^a^
1 %-*ε*-PL-CS/PLA	62.80 ± 0.65^bc^	16.70 ± 1.32^ab^	87.43 ± 4.55^ab^	48.09 ± 3.31^ab^	1.59 ± 0.07^b^	2.10 ± 0.05^a^	30.05 ± 0.04^a^	4.08 ± 0.06^ab^
5 %-*ε*-PL-CS/PLA	64.47 ± 0.60^b^	15.26 ± 0.57^bc^	93.80 ± 8.72^ab^	43.64 ± 3.58^bc^	1.62 ± 0.08^ab^	2.20 ± 0.15^a^	38.82 ± 0.15^c^	11.09 ± 0.12^b^
10 %-*ε*-PL-CS/PLA	67.43 ± 1.39^a^	13.42 ± 1.60^c^	102.12 ± 12.13^a^	38.92 ± 3.24^c^	1.77 ± 0.03^a^	1.73 ± 0.21^b^	57.06 ± 0.08^b^	33.17 ± 0.17^c^

Note: Values are presented as mean ± standard deviation. Different superscripts (a, b, c or d) in the same column indicate significant differences (*p* < 0.05).

#### 3.2.3 Mechanical properties

In practical applications, the mechanical strength of the film can affect its durability during use to a certain extent. The tensile strength (TS) and elongation at break (EB) of the film can demonstrate mechanical strength and flexibility, respectively (Homez-Jara et al., 2018). It was found that in Table 1, the TS and EB of PLA was 21.56 MPa and 82.13 %, respectively, while it was decreased to 18.24 MPa and 80.32 %, respectively when it was coated by CS, which may be due to the photoaging of the substrate film resulted from plasma pretreatment. With increasing of ε-PL content, the TS of ε-PL-CS/PLA film decreased and reduced by about 26 % for 10 %-ε-PL-CS/PLA, compared with 0 %-ε-PL-CS/PLA. That maybe due to the weakening of the hydrogen bonds between chitosan molecules and damage of the homogeneous structure formed during film drying caused by the incorporation of ε-PL, which was consistent with the SEM results. Table 1 also shows that the EB of ε-PL-CS/PLA film was enhanced significantly (*P* < 0.05) with increasing of ε-PL content from 80.32 % for 0 %-ε-PL-CS/PLA to 102.12 % for 10 % ε-PL-CS/PLA composite film. That indicates that ε-PL acts as a plasticizer to improve the flexibility of the film by promoting the interaction between the biopolymer chains (Homez-Jara et al., 2018). Wang et al. (Wang, Liu, Liang, Yuan, & Gao, 2014) found that as the ε-PL content in ε-PL/CS films increased, the tensile strength of the films decreased significantly, while the elongation at break increased significantly.

#### 3.2.4 Water contact angle

As shown in Table 1, the water contact angle of the PLA substrate decreased significantly from 79.72° to 55.05° or lower after coating with CS or ε-PL-CS. This change is primarily attributed to the highly hydrophilic groups—such as hydroxyl (-OH) and amino (-NH₂) groups—which are abundant on the chitosan molecular chains. These groups form a hydrophilic interface with elevated surface energy on the coated surface, thereby significantly enhancing the material's wettability. As the ε-PL content increases, the water contact angle of the composite film shows a continuous decreasing trend. Research by Smrithy et al. (Smrithy et al., 2025) revealed that increased ε-PL content enhances the hydrophilicity of PLA. This phenomenon can be explained at the molecular level: ε-Polylysine, a polypeptide composed of 25–35 l-lysine residues, contains one primary amino group (-NH₂) in each structural unit. These amino groups exhibit high reactivity in forming oriented hydrogen bonds with water molecules. When ε-PL is incorporated into the chitosan matrix, the numerous amino groups on its molecular chains interact with the hydroxyl groups of chitosan to form a denser network of hydrophilic sites (Wang et al., 2014; Yu, Rao, et al., 2019).

#### 3.2.5 Water vapor and oxygen transmittance

As shown in Table 1, the water vapor transmission rate (WVT) of the PLA was 1.52 × 10^−12^ g·m^−1^·s^−1^·Pa^−1^, and it exhibited an upward trend with rising ε-PL concentration, increasing from 1.49 × 10^−12^ g·m^−1^·s^−1^·Pa^−1^ (0 %-ε-PL-CS/PLA) to 1.77 × 10^−12^ g·m^−1^·s^−1^·Pa^−1^ (10 %-ε-PL-CS/PLA). This increase is attributed to the intrinsically hydrophilic nature of ε-polylysine. Being a strongly hydrophilic polypeptide, ε-PL possesses a high density of primary amino groups (-NH₂) along its molecular chain, which endows it with a remarkable capacity to bind water molecules. At elevated ambient humidity, these amino groups capture water molecules via hydrogen bonding, forming localized hydration layers. This property allows ε-PL to act as a “moisture transport channel” within the composite film, facilitating the hopping transfer of water molecules along its molecular chains and thereby significantly enhancing the water vapor diffusion rate through the film (Homez-Jara et al., 2018). Furthermore, the incorporation of ε-PL may disrupt the structural integrity of the chitosan film network during the drying process, which can accelerate water diffusion into the film's interior (Wu et al., 2019). Overall, however, no significant difference in water vapor transmission rates was observed between the pristine PLA and the various PLA-based bilayer films, being consistent with findings by Smrithy et al. (Smrithy et al., 2025), ε-PL enhances water vapor permeability (WVP).

Being diverse to WVP, the OT of PLA was 127.33 cm^3^/m^2^·24h·0.1 MPa. When coated with ε-PL-CS, the OT decreased dramatically to 2.03 cm^3^/m^2^·24h·0.1 MPa (0 %-ε-PL-CS/PLA) and it became lower and lower with increase of ε-PL concentration shown in Table 1, which suggested the oxygen barrier properties of PLA substrate was significantly improved due to dense molecular network structure of chitosan-based coatings (Liu et al., 2022). That is contrary to the findings of Smrithy et al. (Smrithy et al., 2025), the addition of CS may enhance oxygen resistance.

#### 3.2.6 Antibacterial properties

**Fig. 6:**
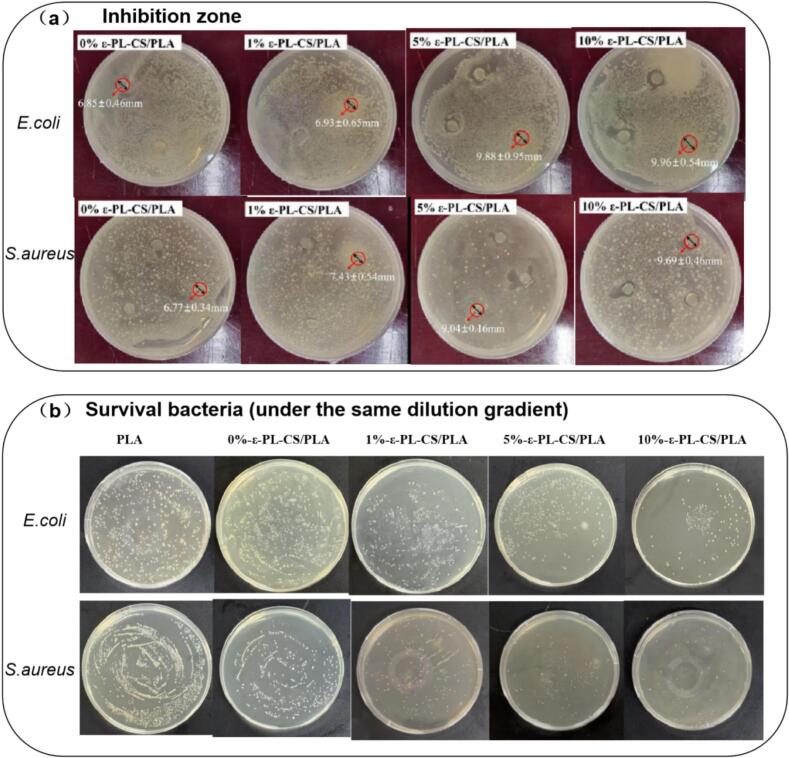
Antibacterial activity of films against *E. coli* and *S. aureus.*

Strong antibacterial properties of packaging films are essential for maintaining food quality to extend its shelf life (Hernández, Ludueña, & Flores, 2023). The antibacterial properties of ε-PL-CS/PLA composite films with different ε-PL contents against *E. coli* and *S. aureus* were shown in Fig. 6. Fig. 6a showed that the inhibition zone of ε-PL-CS/PLA was increasing with the increase of ε-PL concentration, and from 6.85 mm (0 %-ε-PL) to 9.96 mm (10 %-ε-PL) for *E.coli*, and from 6.77 mm (0 %-ε-PL) to 9.69 mm (10 %-ε-PL) for *S. aureus*, respectively, which is consistent with the research results of Wang et al. (Wang et al., 2014). It was similar from Fig. 6b and Table 1 that the antibacterial activity was stronger with the enhancement of ε-PL content resulted from the decrease of survival bacteria with the antibacterial activity increased from 26.96 % to 57.06 for *E. coli*, and from 3.59 % to 33.17 % for *S. aureus*.

Studies showed that the positively charged free amino groups of CS and ε-PL can interact with the negatively charged residues on the bacterial surface, damaging the cell wall structure, resulting in rupture of the outer cell membrane, leakage of intracellular components, and even cell death (Alirezalu et al., 2021; Tuersuntuoheti et al., 2019). Li et al. (Li YaNa et al., 2018) also found that composite films incorporating ε-PL exhibited antibacterial inhibitory effects.

#### 3.2.7 FTIR

**Fig. 7:**
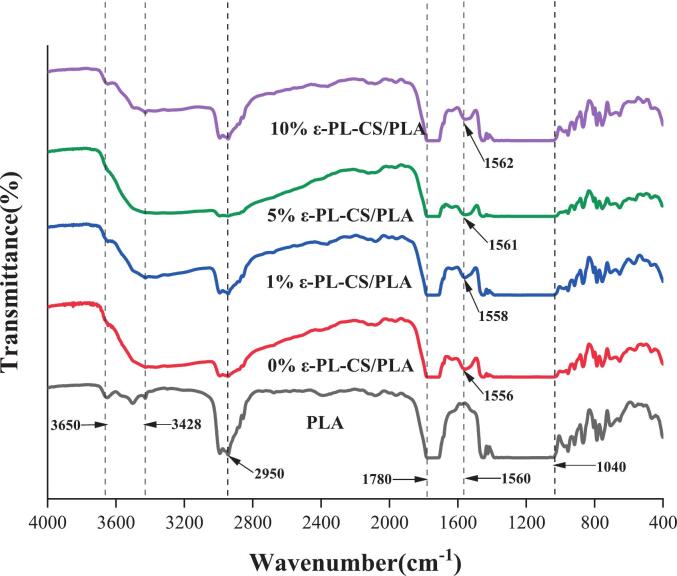
FTIR of PLA or ε-PL-CS/PLA composite films.

The Fourier transform infrared (FTIR) spectrum shown in Fig. 7 provides evidence for the molecular interaction mechanism within the ε-PL-CS/PLA composite film. The characteristic absorption peak at 1780 cm^−1^ in pure PLA corresponds to the stretching vibration of the C=O ester group, while the broad band at 3650–3428 cm^−1^ in CS is attributed to the stretching vibrations of O—H and N—H groups. Upon composite film formation, the significant broadening and red shift of the absorption band in the 3650–3428 cm^−1^ region directly confirms the formation of an extensive hydrogen-bond network between the PLA carbonyl oxygen atom and the N—H and O—H groups in CS and ε-PL (Gupta, Pal, Woo, & Katiyar, 2018). This red shift arises because hydrogen bond formation reduces the force constants of O—H and N—H bonds, shifting their characteristic vibration frequencies toward lower wavenumbers. As ε-PL concentration increases, this spectral broadening becomes more pronounced, indicating that the abundant primary amino groups (-NH₂) in ε-PL molecules act as strong donor sites, establishing additional hydrogen bond crosslinks with the PLA carbonyl acceptor (Yu, Liu, and Huo, 2019). The slight shift in the amide II band absorption peak at 1560 cm^−1^ (primarily corresponding to the N—H bending vibration in CS) further confirms the involvement of CS amino groups in intermolecular interactions. Consistent with the findings of Zhang et al. (Zhang et al., 2015). This shift indicates a redistribution of the N—H bond's electron density following hydrogen bond formation, thereby altering its vibrational characteristics. Concurrently, the C-O-C stretching vibration peak at 1040 cm^−1^ in PLA exhibits corresponding peak shape changes, suggesting the ester bond oxygen atom may participate in intermolecular interactions as a hydrogen bond acceptor (Wang et al., 2022).

### 3.3 Application in pork packaging

#### 3.3.1 pH in pork

**Fig. 8:**
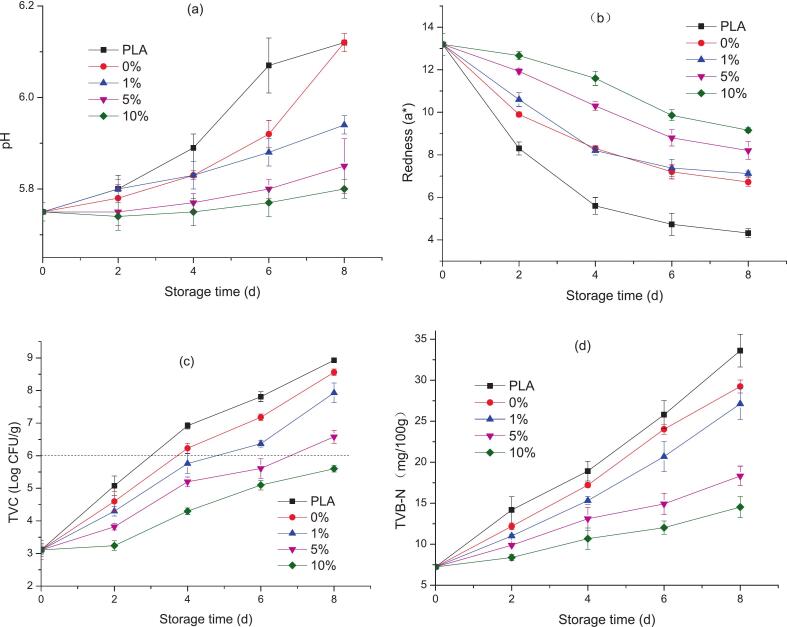
pH, redness, TVC and TVBN of pork packaged with PLA or ε-PL-CS/PLA composite films.

pH value is one of the most critical parameters affecting the microbial balance of meat and evaluating the freshness and quality of fresh meat (Guan et al., 2025). Fig. 8a showed the pH of pork packaged with PLA and ε-PL-CS/PLA composite films during storage time. It was found that from Fig. 8a, the initial pH value of fresh pork before packaging was about 5.75 at 0 d, which was consistent with the literature (Guo et al., 2023; Wang et al., 2022). However, with the extension of storage time, most of the pH of pork increased since some proteins and amino acids in pork are broken down into alkaline substances such as ammonia and amines under the action of bacteria and meat enzymes (Liu et al., 2019), which also was influenced by packaging films. At storage time of 8 d, the pH values of pork packaged by PLA and ε-PL-CS/PLA films with ε-PL concentration of 0 %, 1 %, 5 % and 10 % was 6.12, 6.12, 5.94, 5.85, and 5.80, respectively. That the significant lower growth of pH for pork packaged with a higher ε-PL concentration (5 % and 10 %) of ε-PL-CS/PLA films compared with PLA indicated that films containing ε-PL can inhibit the growth of bacteria, thus postponing the decomposition of protein and amino acids in pork (Zhang et al., 2015).

#### 3.3.2 Redness

During refrigeration at 4 °C, hydrogen sulfide is produced in meat due to microbial activity. The chemical reaction between hydrogen sulfide and myoglobin produces myoglobin sulfide, which causes the meat to turn brown and leads to a decrease in redness value (Liu et al., 2019). According to Fig. 8b, the redness of fresh pork was dramatically decreased for PLA packaging from 13.2 (0 d) to 4.32 (8 d). While for the pork packaged by 0–10 %-ε-PL-CS/PLA films, the decline of redness was delayed with higher redness resulted from higher ε-PL concentration, indicating that the composite films can effectively inhibit the growth of microorganisms in pork, reduce the production of hydrogen sulfide and myoglobin sulfide. Li et al. (Li et al., 2014) discovered that *ε*-PL, through its potent antibacterial action, slows down microbial activity and associated chemical changes that cause myoglobin oxidation, thereby effectively delaying meat color deterioration.

#### 3.3.3 TVC in pork

Meat is rich in nutrients and water, creating a favorable environment for the growth of spoilage microorganisms (Qin et al., 2013). The deterioration of pork can be monitored by the change of total viable count (TVC). According to the safety requirements for pollution-free livestock and poultry meat of Chinese agricultural products safety and quality (GB18406.3–2001), the TVC value of fresh pork cannot exceed 6 Log CFU/g.

The Fig. 8c showed that with the extension of storage time, the increase of TVC for the bilayer films was significantly inhibited compared with PLA. Furthermore, in the same storage time, the TVC was decreasing with the increase of ε-PL content，indicating higher concentrations of ε-PL can better inhibit microbial growth on pork. Fig. 8c also showed that the shelf life of pork packaged by PLA was about 2 d, however it was prolonged to 4–8 d for 1–10 %-ε-PL-CS/PLA. Hence the higher the content of ε-PL, the better the quality protection of pork can be achieved. The antibacterial mechanism of ε-PL is mainly to destroy the cell membrane structure of microorganisms, resulting in the interruption of cell material, energy and information transmission, and ultimately cell death (Wang et al., 2020; Xiao et al., 2025). Guo et al. (Guo, Meng, & Fang, 2017) found the similar results for ε-polylysine/carboxymethyl chitosan polyelectrolyte complex on the packaging of raw pork, showing that ε-PL has bactericidal effects.

#### 3.3.4 TVB-N in pork

Volatile basic nitrogen (TVB-N) is also commonly used as one of the key indicators for evaluating the freshness of pork. The formation of TVB-N is the result of protein degradation, microbial decay, and endogenous enzyme activity, producing volatile nitrogen-containing compounds (Guo et al., 2017). As shown in Fig. 8d, the TVB-N of each group increased with the prolongation of storage time. The TVB-N values of pork packaged with PLA film increased rapidly, while it was delayed for the other groups. Meanwhile, with the increase of ε-PL content, the rise of TVB-N was slower. This is consistent with the results of the TVC of pork. This finding indicates that ε-PL can effectively inhibit bacterial protein degradation, reduce the content of ammonia and amines in pork, and thus delay the increase of TVB-N values in pork. This aligns with the findings of Zheng et al. (Zheng, Tang, Yang, Ran, & Li, 2023) regarding the observed trends of TVB-N in active packaging films based on collagen, tannic acid-modified chitosan, and ε-polylysine, revealing that ε-PL exhibits significant antimicrobial activity in pork packaging.

## 4 Conclusions

To develop composite materials that combine the mechanical advantages of PLA with chitosan based bioactive substances, the pretreatment of PLA surface was studied firstly to improve the compatibility of between PLA and CS. A 5-min air plasma treatment was determined as the optimal condition, which not only significantly improved the hydrophilicity of the PLA surface but also maintained excellent long-term stability. This ensured uniform spreading and strong adhesion of the CS coating, with SEM images revealing a tightly bonded interface between the two layers, thus a bilayer ε-PL-CS/PLA composite film was successfully prepared. The composite films with varying ε-PL contents were systematically evaluated for mechanical properties, water vapor transmission rate (WVT), oxygen barrier rate (OT), water contact angle (WCA), morphology, FTIR characteristics, and antibacterial performance. Results indicate that ε-PL-CS coating improved dramatically the barrier property and endowing antibacterial properties of PLA. For bilayer ε-PL-CS/PLA films, increasing ε-PL content enhances the composite film's elongation at break, oxygen barrier properties, and antibacterial performance, while simultaneously reducing tensile strength and water vapor barrier properties. Incorporation of ε-PL significantly enhances the CS/PLA composite's inhibitory effect against *E. coli* and *S. aureus*, with the 10 % ε-PL-CS/PLA film exhibiting the most pronounced antibacterial activity. When applied to fresh pork packaging, the high ε-PL concentration composite film effectively suppressed increases in pH, TVB-N, and TVC compared to pure PLA film. It minimized the decline in meat color redness and significantly extended pork shelf life from 2 days to a maximum of 8 days. The successful integration of ε-PL into a fully biodegradable PLA/CS system has effectively activated its antimicrobial function, overcoming the performance limitations of conventional biodegradable packaging materials. These findings demonstrate the considerable application potential of the bilayer ε-PL-CS/PLA film in active packaging. Future research should prioritize the enhancement of its tensile strength and moisture barrier properties, as the development of these characteristics remains comparatively inadequate at present.

## CRediT authorship contribution statement

**Li Yana:** Writing – review & editing, Writing – original draft, Investigation, Funding acquisition, Formal analysis, Data curation, Conceptualization. **Zhou Guorui:** Software, Resources, Project administration, Methodology.

## Declaration of competing interest

The authors declare that they have no known competing financial interests or personal relationships that could have appeared to influence the work reported in this paper.

## Data Availability

Data will be made available on request.
